# Nutritional and anti-nutritional compositional analysis of transgenic potatoes with late blight resistance

**DOI:** 10.3389/fbioe.2024.1432079

**Published:** 2024-08-01

**Authors:** Mukani Moyo, Eric Magembe, Lucy Mwaura, Arinaitwe Abel Byarugaba, Alex Barekye, Moses Nyongesa, Catherine Taracha, Marc Ghislain

**Affiliations:** ^1^ International Potato Center, Nairobi, Kenya; ^2^ National Agricultural Research Organisation, Entebbe, Uganda; ^3^ Kenya Agricultural and Livestock Research Organization, Nairobi, Kenya

**Keywords:** potato, transgenic, composition, unintended effect, safety assessment

## Abstract

Late blight, caused by the pathogen *Phytophthora infestans*, is a devastating disease affecting potato production globally, with adverse effects in Africa where limited access to fungicides exacerbates its impact. Outbreaks of late blight lead to reduced yields and substantial economic losses to potato farmers and agricultural systems. The development of resistant potato varieties, tailored to African agroecological conditions, offers a viable solution in mitigating the devastating effects of late blight on potato cultivation. Leading to this study, two consumer-preferred varieties, Victoria and Shangi, with high susceptibility to late blight were targeted for conferring late blight resistance through genetic engineering. This was achieved by inserting *R* genes from wild relatives of potato displaying resistance to the disease. The intended effect of conferring resistance to the late blight disease has been consistently observed over twenty experimental field trials spanning 8 years at three locations in Uganda and Kenya. In this study, we assessed whether the genetic transformation has led to any significant unintended effects on the nutritional and anti-nutritional composition of potato tubers compared to the non-transgenic controls grown under the same agroecological conditions. The compositional assessments were conducted on commercial-size potato tubers harvested from regulatory trials at three locations in Uganda and Kenya. Statistical analysis was conducted using two-way analysis of variance comparing transgenic and non-transgenic samples. Overall, the results showed that the transgenic and non-transgenic samples exhibited similar levels of nutritional and antinutritional components. Variations detected in the levels of the analysed components fell within the expected ranges as documented in existing literature and potato composition databases. Thus, we conclude that there are no biologically significant differences in the nutritional and anti-nutritional composition of transgenic and non-transgenic potato tubers engineered for resistance to late blight.

## Introduction

Potato (*Solanum tuberosum* L.) ranks third in global food crop production after rice and wheat with global total crop production exceeding 300 million metric tons (Devaux et al., 2014). Production and consumption of potatoes have been declining in the developed regions, while in the developing regions, it surpassed the developed regions’ production in 2005 and continue to rise (Devaux et al., 2014; Haverkort and Struik, 2015). Potatoes are consumed by more than a billion people worldwide, making it a critical crop for food security in the face of population growth and increased hunger rates. Potatoes are typically consumed cooked, either from fresh tubers or from processed products that can already be pre-cooked. Consumption of raw tubers is rare due to their unappealing texture, low digestibility, and presence of anti-nutrients and toxicants which are partially destroyed during the cooking process. The consumption of processed potato products is a growing global trend, especially in urban areas where consumers tend to opt for convenient, ready-to-eat foods. Due to the overall importance of potatoes in diets worldwide, the characterization of their nutritional and anti-nutritional properties is crucial for public health (Camire et al., 2009). Though potatoes can also be used for industrial products (starch) and as livestock feed, the primary use in developing countries remains as food (OECD, 2002; OECD, 2021).

Potatoes are rich in carbohydrates, making them an excellent energy source with low fat content. Although low in protein, potatoes also contain a good amount of essential amino acids, dietary and crude fibers, vitamin C, several B vitamins, and potassium. According to Burton (1989) and Ooko (2008), potatoes are a good source of iron (2.5–10 mg/100 g), phosphorus and magnesium (60–140 mg/100 g), copper (0.06–2.83 mg/100 g), calcium (30–90 mg/100 g) and an excellent source of potassium (320–450g/100 g of potato). About 200 g of potatoes provides 10% of the recommended daily intake for phosphorus and magnesium, and up to 20% for copper, iron, and iodine. In addition, approximately 200 g will supply 2–4 g of dietary fiber which is equivalent to about half that supplied by other commonly eaten vegetables (Burton, 1989). Potato tubers have been reported to contain up to 46 mg/100 g ascorbic acid (fresh weight basis) depending on the variety, maturity of the tubers at harvest, and the environmental conditions under which they were grown (Haase and Weber, 2003; Nourian et al., 2003; Han et al., 2004; Burlingame et al., 2009). The variety is the greatest determinant of variation of ascorbic acid concentration in potatoes (Hamouz et al., 2009; Hemavathi et al., 2010).

Apart from the nutritional benefits of potato tubers, they also contain anti-nutrients such as protease inhibitors and lectins, but both are inactivated during cooking. Potato possesses natural toxicants of which the most important are the glycoalkaloids (GA) composed mainly of α-Solanine and α-Chaconine. Together they form 95% of the total glycoalkaloids (TGA) present in potatoes, α -chaconine being present in relatively higher proportions (Friedman, 2006). GA are thought to function in the chemical defense of the plant, as non-specific protectors or repellents against potential pests and predators (Roddick, 1989). In addition, stress tolerance is correlated to GA content (Veilleux et al., 1997). The highest concentration of GA is found within the green parts and in the sprouts. The TGA concentration is high within the peel and just below it and generally decreases with an increase in tuber size. The levels of TGA vary significantly between cultivars (Hellenas, 2001; Friedman, 2006) and are influenced by both genetic and environmental factors as well as various pre- and post-harvest stresses (Dale et al., 1998). TGA in tubers ranging from 20–100 mg/kg or below are of no food safety concerns (FAO/WHO, 1999). Excess levels of GA give potatoes a bitter taste and consumption of such tubers can result in poisoning with several symptoms ranging from gastrointestinal disorders, hallucinations, and partial paralysis to convulsions, coma, and death (Smith et al., 1996). Acrylamide is another anti-nutrient formed in potatoes during high-temperature cooking processes, however, multiple post-harvest strategies have successfully reduced the acrylamide content of processed potato products (Amrein et al., 2003; Liyanage et al., 2021; Kumari et al., 2022). Allergens are not prominent in potatoes, and it is generally considered to be a food source with low, if any, allergenicity potential. A more detailed description of the nutrient, antinutrient, toxicant, and allergen contents of the potato can be found in Camire et al. (2009) and OECD (2002, OECD, 2021).

Leading to the current study, transgenic events were produced by genetic transformation of several varieties by introducing three late blight resistance (*R*) genes and the *nptII* selectable marker gene. The intended effect was to confer resistance to the late blight disease which has indeed been observed in multiple seasons and locations (Ghislain et al., 2019; Webi et al., 2019; Byarugaba et al., 2021). None of these genes and their products are expected to cause changes in the nutritional and anti-nutritional components of the potato. Previous studies evaluated insect and virus-resistant potatoes which were commercially released in the late 90s for compositional analyses, revealing their equivalence to their isogenic non-transgenic counterparts (Rogan et al., 2000; El Sanhoty et al., 2004; Zdunczyk et al., 2005; El-Khishin et al., 2009; Khalf et al., 2010; Chen et al., 2020).

In this study, compositional analysis has been conducted to establish if the genetically engineered plants have significant changes in essential nutritional and anti-nutritional components compared to the plants they are derived from (Codex, 2003). Two types of changes are considered in a food safety assessment of genetically engineered plants: (i) the expected changes resulting from the expression of the new genes added to the plant which are assessed for allergenicity and toxicity specific to the gene products; and (ii) the unexpected changes observed without a clear causal origin. In general, it is assumed that these unexpected changes can arise due to *in vitro* cell and tissue culture, or insertional effects of the T-DNA in the plant genome. Compositional analysis is one method to assess these changes. Hence, if any changes in composition are identified, expected or unexpected, these are then assessed for their potential to cause harm. Worth mentioning, a 20-year review of this approach for safety assessment has revealed that genetic modification has not led to unintended compositional changes (Herman and Price, 2013). This conclusion is supported by extensive evaluation of 148 transgenic events by the U.S. FDA, 189 submissions evaluated by the Japanese regulators and over 80 peer reviewed publications on compositional safety of GM crops. The genetic modifications targeted traits such as drought tolerance, nutrient enhancement, insect resistance, amongst others in a wide range of crops including potato, tomato, cabbage and mushrooms. Additionally, a metabolomics study on five potato cultivars showed that the GM plants exhibited changes in metabolite abundance comparable to conventionally bred varieties (Catchpole et al., 2005).

Compositional analyses were conducted on several transgenic events from two varieties (Victoria, Shangi) to assess whether the gene construct is conducive to unexpected effects and whether any of the transgenic events differ significantly from potatoes recognized as safe for consumption. The study was conducted to ensure compliance with regulatory frameworks set by the national biosafety authorities regulating GM crops in Kenya and Uganda in accordance with the internationally accepted standards for food safety assessment of GM plants established by the Codex Alimentarius (Codex, 2003). A two-step process is used to determine the similarity between the transgenic event and the variety. First, the difference between values for each component is tested statistically for significance. Those found significant are then compared to those from other potato varieties. Should one or more components fall markedly outside the range known from the literature, additional food safety investigations will be advisable.

## Materials and methods

### Plant materials

Two potato varieties produced transgenic events of interest for release in African countries (specifically Kenya, Nigeria, Rwanda, and Uganda). The variety Victoria, also known as Asante in Kenya, has generated three transgenic events, Vic.1, Vic.172, and Vic.185, considered as suitable for release based on molecular characterization and agronomic performance. Vic.1 tubers were obtained from a regulatory trial (confined field trials) at one location in Uganda in 2018, Vic.172 tubers were from trials in three locations in Uganda in 2020 and in 2021 and from three locations in Kenya in 2023, and Vic.185 tubers were from three locations in Kenya in 2023 (Table 1). The variety Shangi, widely grown and popular in Kenya, has generated one transgenic event considered for release in Kenya, Nigeria and Rwanda. Sha.105 tubers were obtained from confined field trials at three locations in Kenya (Table 1).

**TABLE 1:** Transgenic potato events and non-transgenic varieties, years and locations of trials, number of replications (blocks) per trial, number of samples per replicate, and total sample number (N) used for compositional analysis.

Potato variety	Transgenic event	Year	Location of confined field trials	Replicates per trial	Samples per replicate	Total sample (N)
Victoria	Vic.1	2018	Uganda: Kashwekano	4	2	8
	Vic.172	2020	Uganda: Kashwekano, Rwebitaba, Buginyanya	3	2	18
		2021	Uganda: Kashwekano, Rwebitaba, Buginyanya	3	2	18
		2023	Kenya: Muguga, Njabini, Molo	1–3^a^	1	8 (7)
	Vic.185	2023	Kenya: Muguga, Njabini, Molo	2–3^a^	1	8
Shangi	Sha.105	2023	Kenya: Muguga, Njabini, Molo	2–3^a^	1	8

^a^ In the 2023 Kenya Trials: one sample was collected from three replicates at two sites and from two replicates at one site, for Victoria, Vic. 185, Shangi and Sha.105 (N = 8); one sample was collected from three replicates at two sites and from one replicate at one site for Vic.172 (N = 7).

### Sampling tubers for compositional analysis

Tubers from the transgenic event(s) and the comparator (the non-transgenic variety it derives from) were harvested from each location and samples collected for compositional analysis. The design of each confined field trial was a randomized complete block design with three blocks (replications) (Byarugaba et al., 2021) except the Vic.1 trial in 2018 Kashwekano, Uganda where there were four replications (Table 1). The total number of samples for each genotype combined across trial sites are shown in Tables 1, 2 and indicated in parenthesis in the results section (Tables 4–8). At harvest, for each sample, four medium-sized tubers, each weighing at least 100g, were selected as free of any defect (no regrowth, no mechanical injuries, no damage due to pests or diseases) and clean of any soil.

**TABLE 2:** Sample sizes and nutritional components measured for Event Vic.172 and Victoria collected from trials in Uganda 2020 following OECD (2002) recommendations and Uganda 2021 and Kenya 2023 following OECD (2021) recommendations.

Component		Uganda 2020	Uganda 2021	Kenya 2023	Total
Moisture	Vic.172	18	18	7	43
	Victoria	18	18	8	44
Protein	Vic.172	18	18	7	43
	Victoria	18	18	8	44
Total Sugars	Vic.172	18			18
	Victoria	18			18
Fat	Vic.172		18	7	25
	Victoria		18	8	26
Carbohydrate	Vic.172		18	7	25
	Victoria		18	8	26
Ash	Vic.172		18	7	25
	Victoria		18	8	26
Dietary Fiber	Vic.172		18	7	25
	Victoria		18	8	26
Starch	Vic.172		18	7	25
	Victoria		18	8	26
Vitamin C	Vic.172	18	18	7	43
	Victoria	18	18	8	44
Vitamin B6	Vic.172		18	7	25
	Victoria		18	8	26
Potassium	Vic.172		N/A	7	7
	Victoria		N/A	8	8
Magnesium	Vic.172		18	7	25
	Victoria		18	8	26
Total Glycoalkaloids	Vic.172	18	18	7	43
	Victoria	18	18	8	44

N/A not available.

Tubers from Kenya and Uganda were approximately 1-month and two-month-old, respectively, before being processed by the International Potato Center’s (CIP) Food And Nutritional Evaluation Laboratory (FANEL) at the Bioscience for east and central Africa (BecA) at ILRI in Nairobi, Kenya. The age difference is due to the process for handling and exporting these transgenic tubers over borders since they are considered as restricted materials and thus classified and handled following UN3245 norms. Movement of this material was fully compliant with biosafety and phytosanitary regulations in place for both countries.

All fresh samples were kept at room temperature until use. Samples were coded and handed over to the FANEL team without the key code that provides the full identity of the sample. Samples were screened for morphological anomalies, for any damage resulting from pest, disease, and handling. In all cases, tubers were peeled before use. Tubers from the same sample were pooled together and three technical replicates taken for all analyses. Results are presented on fresh weight basis. All lyophilized samples were milled to a fine powder and kept at −80°C for the duration of analyses to preserve sample quality.

The samples for Vic.1 from one trial site harvested in 2018 (Table 1) were analyzed for five components as recommended in OECD (2002) (Table 4). The samples for Vic.172 were from three trial sites and 3 years (Table 1). Samples from three sites harvested in 2020 were analyzed for the five components in OECD (2002) and analyzed for 12 components as recommended in OECD (2021) from three sites harvested in 2021 and in 2023 (Table 2). The samples from Vic.185 from three sites harvested in 2023 (Table 1) were analyzed for 12 components as in OECD (2021) (Table 6).

### Determination of moisture content

The method is based on drying the sample under controlled temperature conditions until a constant weight is obtained as per the method described by AOAC (2012b).

### Determination of sugar (reducing and total) content

The quantification of individual and total sugars in potato tubers was done according to the method described by Sesta (2006). Briefly, sugars were extracted from lyophilized potato material using 85% ethanol. The ethanol was then evaporated, leaving the sugars in an aqueous solution. The individual sugars were separated on the HPLC using a Eurospher 100–5 NH2 column (Knauer, Berlin) and detected by a refractive index detector.

### Determination of protein content

Protein content was determined using the Kjeldahl method as described by AOAC (2012b). The analysis involves the digestion of potato samples in sulfuric acid in the presence of a Kjeldahl tablet, containing catalysts that facilitate the release of nitrogen from protein-bound nitrogen and free amino acids. The amount of nitrogen is then used to calculate crude protein content.

### Determination of fat content

Fat and oils (total fat) were determined by hydrolyzing 3 g of the lyophilized samples containing 1 g of Celite using 4N HCl followed by solvent extraction using petroleum ether as per AOAC (2012b).

### Determination of ash content

Ash content was determined by AOAC (2012b) method. The method involves the oxidation of the organic matter in potato samples in a furnace at 550°C for at least 4 h.

### Quantification of carbohydrates

Carbohydrate content was obtained by calculation by removing moisture, protein, fat, and ash content in %.

### Determination of dietary fiber

Dietary fiber was determined according to AOAC (2012b). The method involves the sequential digestion of lyophilized potato samples with dilute acid and alkali solutions, leaving behind the indigestible components that make up the dietary fiber content.

### Determination of total starch

This was measured using the Total Starch Assay kit (Megazyme, Ltd), as per manufacturer instructions.

### Determination of vitamin C

The method detects vitamin C (ascorbic acid) using the HPLC method as described in Gazdik et al. (2008). The lyophilized potato samples are homogenized in 3% metaphosphoric acid before being filtered and chromatographed using the HPLC.

### Determination of vitamin B6

The extraction of Vitamin B6 was done as per the method in Zand et al. (2012). The lyophilized potato samples were solubilized and Vitamin B6 was extracted using 1% acetic acid, heated at 70°C for 40 min in a water bath. The solutions were centrifuged and filtered before analysis using the HPLC.

### Determination of minerals: Potassium (K) and magnesium (Mg)

The mineral analysis was done by ICP-OES equipment, Perkin Elmer, Optima 2100. 0.3 g of the sample was digested in nitric acid and hydrogen peroxide at 150°C in a microwave digestor for 20 min. The solution was cooled and diluted to 20 mL with 1% nitric acid and the minerals values were read out in the ICP-OES machine using the WinLab software. The calibration curves in a range of 0–2 mg/L were developed using the standards and blanks from Perkin Elmer.

### Determination of glycoalkaloids

The glycoalkaloids were extracted as per the method by Tomoskozi-Farkas et al. (2006). Extraction is done on lyophilized potato samples using dilute acetic acid. The extract is concentrated and cleaned up on disposable solid-phase extraction cartridges. The final separation and measurement of α-solanine and α-chaconine was done by HPLC (AOAC, 2012a).

### Literature range of potato components

The OECD (2021) used an extensive survey of publications, private and national database to establish ranges among conventional varieties of potato for the components of the study. These ranges were broader than those from AFSI (2024). We expanded most of them using data from other publications not included in the OECD survey (Burlingame et al., 2009; Dhingra et al., 2012; Chen et al., 2020; Tatarowska et al., 2023). The new ranges were established by using the lower and upper limits from these publications (Table 3).

**TABLE 3:** Ranges for the components analyzed using literature on a fresh weight basis.

Component	Literature range	Units	References
Moisture	63–87	%	Burlingame et al. (2009)
Protein	0.85–4.2	%	Burlingame et al. (2009)
Total sugar	0.05–8	%	OECD (2002)
Fat	0.00–0.30	%	OECD (2021)
Carbohydrates	12.9–19.6	%	AFSI, 2024; Chen et al., 2020
Ash	0.62–1.36	%	AFSI (2024)
Dietary fiber	1.30–3.6	%	Dhingra et al., 2012; OECD, 2021
Starch	7.3–22.6	%	Burlingame et al., 2009; Chen et al., 2020
Vitamin C	1.0–42	mg/100 g	Burlingame et al., 2009; Tatarowska et al., 2023
Vitamin B6	0.11–0.26	mg/100 g	Chen et al., 2020; OECD, 2021
Potassium	239–693.8	mg/100 g	Burlingame et al. (2009)
Magnesium	7.7–37.6	mg/100 g	Burlingame et al., 2009; OECD, 2021
Total glycoalkaloids	0.071–175	mg/100 g	Burlingame et al. (2009)

### Statistical analysis

The analysis of variance was made assuming the effects of Genotypes, Locations, and the interaction of genotypes with locations, such as fixed effects, to the effect of blocks within locations, such as random effects. We used the ANOVA: Two-Factor with Repetition function of the Analysis ToolPak Excel for Microsoft 365 except for Vic.172 which combined 3 year data with different numbers of samples. In this case, we used Tukey’s honesty significant difference (HSD) using R statistical package (version 4.4.1) to obtain the *p* values. Significance was declared at *p* < 0.05.

## Results

### Transgenic events from the variety Victoria

Three transgenic events derived from the variety Victoria were analyzed using the five or the 12 components recommended by OECD 2002; OECD, 2021 respectively.

The five components analyzed in tubers from Vic.1 and its comparator, the variety Victoria, had essentially the same value and the small differences were not statistically significant (Table 4). Moisture was quite high, around 82% for both genotypes but typically within the literature range. Protein content was around 2 g/100 g for both genotypes which is the most commonly reported value in potato tubers. Total sugar of 0.36 and 0.51 g/100 g for Vic.1 and Victoria respectively was also within the literature range. Vitamin C of 7.44 and 6.22 mg/100 g was also within the literature range and the differences were not statistically significant. Finally, total glycoalkaloids were 0.31 and 0.36 mg/100 g for Vic.1 and Victoria, a relatively low value compared to other potato varieties but within the literature range.

**TABLE 4:** Compositional analysis of tubers of Vic.1 *versus* Victoria from Uganda in 2018 following OECD (2002).

Component	Vic.1		Victoria		*p*-Value	Literature range	Units
	Mean(N)	Range	Mean(N)	Range			
Moisture	82.14 (8)	78.38–84.89	81.62 (8)	78.23–83.75	0.599	63–87	%
Protein	2.04 (8)	1.80–2.34	2.026 (8)	1.68–2.60	0.930	0.85–4.2	%
Total sugars	0.36 (8)	0.31–0.42	0.51 (8)	0.26–1.00	0.124	0.09–4.3	%
Vitamin C	7.44 (8)	4.62–11.54	8.48 (8)	6.22–11.86	0.319	2.8–42	mg/100 g
Total glycoalkaloids	0.305 (8)	0.082–0.994	0.364 (8)	0.083–0.688	0.701	0.071–175	mg/100 g

(N) = total number of samples.

Tubers from the transgenic event Vic.172 were analyzed separately on three occasions (Table 1, 2), once with the OECD (2002) components and twice with the OECD (2021) components. This transgenic event was also assessed for its agronomic performance (Byarugaba et al., 2021). The 13 components analyzed on tubers from Vic.172 and Victoria were similar between the two genotypes except for three (Table 5). Ash, vitamin C, and total glycoalkaloids were higher for Vic.172 compared to Victoria and the differences were statistically significant (*p* < 0.05). However, Vic.172 values were still within the literature range.

**TABLE 5:** Compositional analysis of tubers of Vic.172 *versus* Victoria from Uganda in 2020 following OECD (2002), in 2021 following OECD (2021), and from Kenya in 2023 following OECD (2021).

Component	Vic.172		Victoria		*p*-Value	Literature range	Units
	Mean(N)	Range	Mean(N)	Range			
Moisture	76.24 (43)	70.41–80.57	76.75 (44)	70.35–82.83	0.354	63–87	%
Protein	2.49 (43)	1.71–4.94	2.38 (44)	1.44–5.12	0.547	0.85–4.2	%
Total sugars	1.28 (18)	0.61–2.46	1.27 (18)	0.68–1.88	0.967	0.05–8	%
Fat	0.02 (25)	0.01–0.05	0.03 (26)	0.01–0.06	0.064	0.0–0.3	%
Carbohydrates	19.47 (25)	16.61–24.23	19.32 (26)	14.75–23.49	0.778	12.9–19.6	%
Ash	1.05 (25)	0.77–1.34	0.96 (26)	0.68–1.29	0.043^a^	0.62–1.36	%
Dietary fiber	1.98 (25)	1.51–2.97	2.07 (26)	1.40–3.50	0.463	1.30–3.6	%
Starch	12.73 (25)	10.28–16.08	12.44 (26)	9.45–14.70	0.481	7.3–22.6	%
Vitamin C	3.84 (43)	0.14–11.14	2.98 (44)	1.01–11.79	0.131	1.0–42	mg/100 g
Vitamin B6	0.83 (25)	0.01–1.60	0.42 (26)	0.01–1.24	0.005^a^	0.11–0.26	mg/100 g
Potassium	383.71 (7)	244–491	338.5 (8)	228–469	0.328	239–693.8	mg/100 g
Magnesium	18.02 (25)	14.57–23.13	16.64 (26)	9.48–22.28	0.204	7.7–37.6	mg/100 g
Total glycoalkaloids	11.28 (43)	0.42–2.98	7.66 (44)	0.17–1.83	0.007^a^	0.071–175	mg/100 g

^a^ *p* < 0.05; (N) = total number of samples.

The third transgenic event from the variety Victoria, Vic.185, was analyzed for its 12 components from tubers grown in Kenya (Table 1). The results support the conclusion that none of the differences were statistically significant (Table 6). All component values were within the literature range except for protein which was higher for Vic.185 than the literature range but not statistically significantly higher than Victoria (*p*-value of 0.117).

**TABLE 6:** Compositional analysis of tubers of Vic.185 *versus* Victoria from Kenya in 2023 following OECD (2021).

Component	Vic.185		Victoria		*p*-Value	Literature range	Units
	Mean(N)	Range	Mean(N)	Range			
Moisture	75.03 (8)	67.82–82.34	76.27 (8)	73.40–77.20	0.376	63–87	%
Protein	4.64 (8)	3.05–5.23	3.91 (8)	2.56–5.12	0.117	0.85–4.2	%
Fat	0.02 (8)	0.01–0.04	0.03 (8)	0.01–0.04	0.400	0.0–0.3	%
Carbohydrates	19.36 (8)	13.96–25.23	18.78 (8)	16.62–21.23	0.607	12.9–19.6	%
Ash	1.12 (8)	0.65–1.70	1.02 (8)	0.68–1.29	0.267	0.62–1.36	%
Dietary fiber	2.42 (8)	1.77–3.58	2.50 (8)	1.89–3.50	0.761	0.39–3.6	%
Starch	12.77 (8)	9.24–16.12	12.00 (8)	10.84–14.02	0.276	7.3–22.6	%
Vitamin C	5.07 (8)	1.43–11.03	4.75 (8)	1.01–11.79	0.774	2.8–42	mg/100 g
Vitamin B6	0.24 (8)	0.02–0.67	0.11 (8)	0.03–0.26	0.102	0.11–0.26	mg/100 g
Potassium	393 (8)	241–612	339 (8)	228–469	0.125	239–693.8	mg/100 g
Magnesium	14.72 (8)	11.61–22.42	11.90 (8)	9.48–15.49	0.054	7.7–37.6	mg/100 g
Total glycoalkaloids	1.21 (8)	0.25–2.48	0.91 (8)	0.45–1.45	0.251	0.071–175	mg/100 g

(N) = total number of samples.

### Transgenic event from the variety Shangi

One transgenic event from the variety Shangi (Sha.105) was analyzed for the 12 components recommended by OECD (2021). Tubers from Sha.105 were obtained from three locations in Kenya (Table 1). Small differences in the values of the 12 components were observed but none were statistically significant (Table 7). All of these values were within the literature range. Moisture for both Sha.105 and Shangi was lower compared to the previously analyzed genotypes whereas starch and carbohydrate seemed higher. This prompted us to compare the two varieties since tubers were harvested from the same trials. Interestingly, out of the 12 components, the differences of three components (moisture, carbohydrate, and starch) were statistically significant (Table 8).

**TABLE 7:** Compositional analysis of tubers of Sha.105 *versus* Shangi from Kenya in 2023 following OECD (2021).

Component	Sha.105		Shangi		*p*-Value	Literature range	Units
	Mean(N)	Range	Mean(N)	Range			
Moisture	72.76 (7)	63.01–84.57	71.84 (8)	64.67–77.10	0.727	63–87	%
Protein	3.91 (7)	2.35–5.21	4.01 (8)	2.81–5.31	0.822	0.85–4.2	%
Fat	0.03 (7)	0.01–0.05	0.02 (8)	0.01–0.06	0.512	0.0–0.3	%
Carbohydrates	23.14 (7)	12.30–31.04	22.99 (8)	19.02–29.39	0.938	12.9–19.6	%
Ash	1.05 (7)	0.65–1.58	1.14 (8)	0.58–1.64	0.508	0.62–1.36	%
Dietary fiber	2.56 (7)	1.17–4.08	2.78 (8)	2.26–3.39	0.459	0.39–3.6	%
Starch	14.48 (7)	9.12–20.45	15.53 (8)	11.50–18.84	0.505	7.3–22.6	%
Vitamin C	6.74 (7)	1.47–12.66	4.95 (8)	1.52–10.10	0.137	2.8–42	mg/100 g
Vitamin B6	0.10 (7)	0.04–0.20	0.13 (8)	0.02–0.40	0.414	0.11–0.26	mg/100 g
Potassium	359 (7)	236–483	383 (8)	229–513	0.540	239–693.8	mg/100 g
Magnesium	11.53 (7)	8.62–16.52	12.61 (8)	8.51–24.68	0.496	7.7–37.6	mg/100 g
Total glycoalkaloids	0.80 (7)	0.36–1.76	1.04 (8)	0.56–2.19	0.206	0.071–175	mg/100 g

(N) = total number of samples.

**TABLE 8:** Compositional analysis of tubers of Victoria *versus* Shangi from Kenya following OECD (2021).

Component	Victoria		Shangi		*p*-Value	Literature range	Units
	Mean(N)	Range	Mean(N)	Range			
Moisture	76.27 (8)	73.40–77.20	71.84 (8)	64.67–77.10	0.009^a^	63–87	%
Protein	3.91 (8)	2.56–5.12	4.01 (8)	2.81–5.31	0.802	0.85–4.2	%
Fat	0.03 (8)	0.01–0.04	0.02 (8)	0.01–0.06	0.879	0.0–0.3	%
Carbohydrates	18.78 (8)	16.62–21.23	22.99 (8)	19.02–29.39	0.004^a^	12.9–19.6	%
Ash	1.02 (8)	0.68–1.29	1.14 (8)	0.58–1.64	0.171	0.62–1.36	%
Dietary fiber	2.50 (8)	1.89–3.50	2.78 (8)	2.26–3.39	0.175	0.39–3.6	%
Starch	12.00 (8)	10.84–14.02	15.53 (8)	11.50–18.84	0.002^a^	7.3–22.6	%
Vitamin C	4.75 (8)	1.01–11.79	4.95 (8)	1.52–10.10	0.849	2.8–42	mg/100 g
Vitamin B6	0.11 (8)	0.03–0.26	0.13 (8)	0.02–0.40	0.600	0.11–0.26	mg/100 g
Potassium	339 (8)	228–469	383 (8)	229–513	0.109	239–693.8	mg/100 g
Magnesium	11.90 (8)	9.48–15.49	12.61 (8)	8.51–24.68	0.617	7.7–37.6	mg/100 g
Total glycoalkaloids	0.91 (8)	0.45–1.45	1.04 (8)	0.56–2.19	0.451	0.071–175	mg/100 g

^a^ *p* < 0.05; (N) = total number of samples.

## Discussion

Compositional analysis of tubers from transgenic events and the variety they are derived from was conducted to assess unintended compositional effects in the transgenic events following the recommended components by OECD (2002, OECD, 2021). The plant materials were produced from regulatory trials with up to three repetitions, and up to three locations per country. The compositional analyses revealed small differences between the transgenic events and the variety they derived from. For three transgenic events, Vic.1, Vic.185, and Sha.105, none of these differences were statistically significant. For one transgenic event Vic.172, eight of the 12 components were higher than those of Victoria and statistically different for three of them. Still, all component values were within the range of values published in the literature and database. Such small differences with statistical significance between a transgenic event and the variety it derives from have been observed previously but values stayed within the literature range (Rogan et al., 2000; Khalf et al., 2010; Chen et al., 2020).

Our results align with those of other compositional analyses comparing transgenic events with their near isogenic lines demonstrating that there are no unintended biologically significant differences in the nutritional composition between the transgenic events and the non-transgenic variety due to the addition of new genes through genetic transformation. Moisture ranged between 72% and 82%, which is within the literature range of 63%–87% (Burlingame et al., 2009). Protein content was close to 2 g/100 g for the samples from Uganda whereas samples from Kenya were closer to 4 g/100 g. This difference might be related to differences in soil nitrogen content as observed in potato tubers (Rosyidah et al., 2021). Fat for all genotypes was on the lower end of the range for other varieties. Indeed OECD (2021) reports that potato tubers are generally recognized as naturally very low in fat (undetectable to 0.3 g/100 g). Carbohydrates, ash, dietary fiber, and starch were within the range reported in the literature. Vitamins C and B6 were in the low end of the literature range for each genotype. Vitamins are components which may have been underestimated in our analyses due to various factors. During preparation and processing of tubers, water soluble vitamins may be washed out, and/or partially destroyed by heat and oxidation. Ascorbic acid content may also decrease during storage and be affected by environmental conditions (OECD, 2002; Burgos et al., 2009; OECD, 2021). Minerals, potassium, and magnesium were always within the literature range with magnesium being in the lower end of the literature range. Finally, total glycoalkaloids were also in the low end of the literature range. For this component, the low values might be related to the peeling depth. Indeed, the bulk of glycoalkaloids is produced and accumulates within 1.5 mm below the skin (Valkonen et al., 1996).

When the two varieties, Victoria and Shangi, were compared, the differences were significant only for starch and carbohydrates. These components are related to traits breeders focus on. The number one trait for potato breeders is yield. Dry matter content is always assessed because it determines the essential qualities for consuming potatoes as fresh food or processed products. Hence, these differences may be the result of different breeding priorities. Potato varieties may also have differences in their composition in relation to the germplasm it derives from. Cultivated tetraploid potatoes have two germplasm origins, one of the Andigena type well adapted to tropical latitudes and short-day conditions, and the other of Tuberosum type well adapted to temperate latitudes and long day conditions (Spooner et al., 2014). Victoria and Shangi varieties are of the Andigena type whereas most of the compositional data published in databases and the literature are from potato varieties of the Tuberosum type.

## Conclusion

The compositional analyses conducted on tubers grown from several locations, years, countries, and varieties reveal that there were no biologically significant differences in nutritional and anti-nutritional components between the transgenic events and the variety they derived from. In a few cases where differences were observed, the values were within the range reported in literature and compositional databases. The transgenic events, therefore, did not result in unintended compositional changes to the variety they derived from and should be regarded as safe as any other non-transgenic potato variety. Our study also supports the findings of the 20-year review of the use of compositional analysis to detect unintentional changes in transgenic plants, indicating that the introduction of the new genes of our gene construct did not prompt unintended effects (Herman and Price, 2013). Ten years after that study, it is also our firmly held belief that compositional analysis going beyond the standard parameters potato breeders typically evaluate (dry matter and total glycoalkaloids) is not scientifically justified.

## Data Availability

The original contributions presented in the study are included in the article/Supplementary Material, further inquiries can be directed to the corresponding author.
